# Special Issue “Sustainable Asphalt Pavements: Materials, Design Methods, and Characterization Techniques” (First and Second Volumes)

**DOI:** 10.3390/ma15217649

**Published:** 2022-10-31

**Authors:** Orazio Baglieri, Pier Paolo Riviera

**Affiliations:** Department of Environment, Land and Infrastructure Engineering, Politecnico di Torino, 24 C.so Duca Degli Abruzzi, 10129 Turin, Italy

A sustainable approach in asphalt pavement engineering should focus on materials, design methods, and technologies that can contribute to minimizing environmental impacts through a reduction in energy consumption and natural resources, while ensuring that all performance standards and requirements are met.

The decision-making process should be appropriately updated by adopting suitable tools and methodologies, such as LCCA and LCA, to measure the potential economic and environmental impacts associated with the adoption of sustainable solutions.

This Special Issue includes recent and original studies that address the design and construction of sustainable asphalt pavements performed by several researchers belonging to different universities. The scope of the presented studies is extensive and multidisciplinary, covering many aspects related to (i) recycled and renewable materials for the production of asphalt concrete, (ii) low-energy and low-emission technologies, (iii) long-life design solutions, and (iv) green urban development.

(i) Recycled and renewable materials in asphalt pavements may be employed for the modification of asphalt binders and/or for the full or partial replacement of natural aggregates and mineral filler in asphalt mixtures [1,2]. In the study of [3], various asphalt mixtures containing construction and demolition waste (CDW), jet grouting waste (JGW), fly ash (FA), and reclaimed asphalt (RA) were evaluated and compared based on a life cycle impact assessment (LCIA) that follows a “from cradle to grave” approach. Based on LCIA results, the solution involving a cold, in-place recycled mixture constituted by RA and JGW showed the best performance in terms of both service life and environmental effects. A critical point in the use of RA is associated with the presence of reclaimed aged asphalt binder (RAB) that may compromise the fatigue resistance of a mixture, especially when RA is added at higher rates. The impact of RAB on the service life of pavements was investigated in [4]; the experimental data obtained from this study revealed that the inclusion of up to 40% RAB may lead to increased fatigue life of mixture.

(ii) Cold-mix asphalt technologies, including water-foamed asphalt and emulsion-based mixtures, have been extensively used for their energy-saving and emission-reducing features. The study of [5] focused on the mechanism of viscosity reduction in water-foamed asphalt and its impact on mix workability. The results show that moisture evaporation is significantly influenced by the foaming water content and ambient temperature, which results in a different stabilizing time for water-foamed asphalt. The viscosity reduction of asphalt, in turn, is strongly related to the initial foaming water content. An emulsion-based, cold-recycled mixture to be employed as a sustainable solution for the surface finishing of unpaved rural roads was investigated in the study of [6]. It was found that, by means of a purposely fine-tuned mix design procedure, optimal dosages of the recycled components can be identified, thereby ensuring desirable properties in terms of high workability and adequate stiffness and strength.

(iii) Durability and performance life are recognized to be critical factors in the sustainability of road pavements [7]. The Special Issue contains research presenting new ideas and technical proposals that may contribute to the construction of durable and long-lasting pavement structures.

Composite admixtures of lignin and glass fibers have been successfully used as additives to improve the performance of asphalt mixtures, in terms of moisture susceptibility and thermal cracking resistance [8]. The environmental impact of composite mixtures containing lignin and glass fibers was studied in [9]. Based on the data obtained from LCA analysis, it was found that the drawbacks related to the addition of admixtures is minimal with respect to the overall environmental impact of asphalt mixture. Therefore, it was argued that the composite asphalt mixture can be effectively employed to improve the performance of pavements. A high-viscosity hybrid-modified asphalt binder, containing styrene–butadiene–styrene (SBS) combined with a crumb rubber modifier (CRM), was considered in the study of [10] to enhance the durability of an open-graded friction course (OGFC). In fact, the hybrid modification of SBS and CRM can reduce the problem of segregation, contextually increasing the viscosity of the modified asphalt binder. By comparing the performance of various binders based on Zero Shear Viscosity (ZSV), grey relational analysis showed the highest correlation with an anti-rutting indicator for a high-viscosity-modified asphalt binder, thus indicating its pronounced anti-rutting potential. The study of [11] investigated a technical solution specifically developed to reduce the rutting and cracking risks of a bridge deck pavement. The solution consists of pre-coated aggregates grouting asphalt concretes (PGAC) prepared with selected aggregates and various types of polymer-modified binders. The test results show superior mechanical properties for PGAC with the grouting material of a high-viscosity-modified asphalt binder blended with mineral filler.

(iv) The construction of a sponge city is a major green innovation that can be used implement the concept of sustainable development. The study of [12] addresses a permeable asphalt concrete (PAC) pavement for urban areas characterized by pronounced water permeability and noise reduction capability. In particular, the study investigated the use of a two-fold modification technique based on the combination of styrene–butadiene–styrene (SBS) and crumb rubber (CR) to enhance pavement performance. The results highlight that the addition of an optimum content of SBS and CR, not exceeding 15%, slightly reduced the permeability coefficient of PAC, but significantly improved its high-temperature performance, fatigue life, and rutting resistance, as well as resilient modulus.

The editors give special thanks to the authors and the editorial team of Materials for the collaborative and peer-review process. We hope you enjoy reading this Special Issue and find new concepts for future and present research.
